# Supplementary material for: Large-area, high-resolution characterisation and classification of damage mechanisms in dual-phase steel using deep learning
Source: PLoS One. 2019 May 8;14(5):e0216493. doi: 10.1371/journal.pone.0216493 (PMC6505961; doi:10.1371/journal.pone.0216493)
Supplement: S1 Supporting information — This supporting information contains a more detailed description of the network and relevant terms in Table 2 of the main document, the accuracy and loss for the first and second classifier as a function of epoch with the confusion matrices for the test data, a comparison of different networks tested for this work and a comment on validation and hyperparameters. (PDF) [file pone.0216493.s001.pdf]

# - Supporting Information -

Large-area, high-resolution characterisation and classification of damage mechanisms in dual-phase steel using deep learning

Carl Kusche[1,+], Tom Reclik[1,+], Martina Freund[1], Talal Al-Samman[1], Ulrich Kerzel[1,2], Sandra Korte-Kerzel[1,*]

[1] Institute of Physical Metallurgy and Metal Physics, RWTH Aachen University, Aachen, Germany

[2] IUBH University of Applied Sciences, Bad Honnef, Germany

[+] equal contribution

[*] korte-kerzel@imm.rwth-aachen.de

Description of network and relevant terms in Table 2 of the main document

The convolutional layer implements the convolution operator as described in the main document. This allows the network to essentially learn to behave as image filters and extract relevant features from the image automatically. The first convolutional layer in the network takes the micrograph image as input which is characterised by the number of pixels I x and y direction as well as the number of channels. In our case of grayscale images, the number of channels is 1. The window size of the convolutional filter effectively determines which part of the image the filter currently works on and how large an area the convolutional neuron is sensitive to. As this window is by definition smaller than the whole micrograph image, the convolutional filter needs to be moved across to capture the details of the whole image. The stride controls the step size in each direction in which the window is moved. Care should be taken to ensure that the stride is at most the same size as the convolutional window to make sure that the image is fully covered. As the convolutional window approaches the edges of the image, part of the window may fall out of the active image area and new inputs need to be defined for this case. This process is called padding; most popular choices are "zero padding" where the border of the image is filled with zeros or "same padding" where the border is filled with the outermost values of the image to make sure the convolutional window receives a valid input.

Pooling layers are used to control the size of deep neural networks. In particular, convolutional deep neural networks can become very large when many convolutional layers with multiple channel per layer are combined. Pooling layers are used to down-sample the output of one layer before passing it on as input to the next layer. The most popular approach is the so-called "max pooling" where only the maximum value of a specified part of the output of one network layer is used as input for the next, hence removing all but one values from the output.

Dropout was introduced as a regularisation technique for complex neural networks. During training, a fraction of the neurons and their connections are removed randomly. This effectively creates an ensemble of slightly different networks when used for the image classification.

Summary:

Convolution layer: The convolutional layer implements the convolution operator. This allows the network to essentially learn to behave as image filters and extract relevant features from the image automatically.

Pooling layers: These are used to control the size of convolutional neural networks and can become very large when many convolutional layers with multiple channels per layer are combined.

Dropout layer: This is introduced as a regularisation technique for complex neural networks.

Window size: The window size of the convolutional filter effectively determines the image part currently processed by the filter and the size of the image area the convolutional neuron is sensitive to.

Stride: The stride controls the step size in each moving direction of the image window

Channel: Used as a network input within the micrograph image. For grayscale images (current case), the number of channels is 1.

Network performance: Accuracy and loss

Accuracy and loss as a function of epoch with the confusion matrices for the test data.

Fig A: Accuracy and loss for the first classifier.

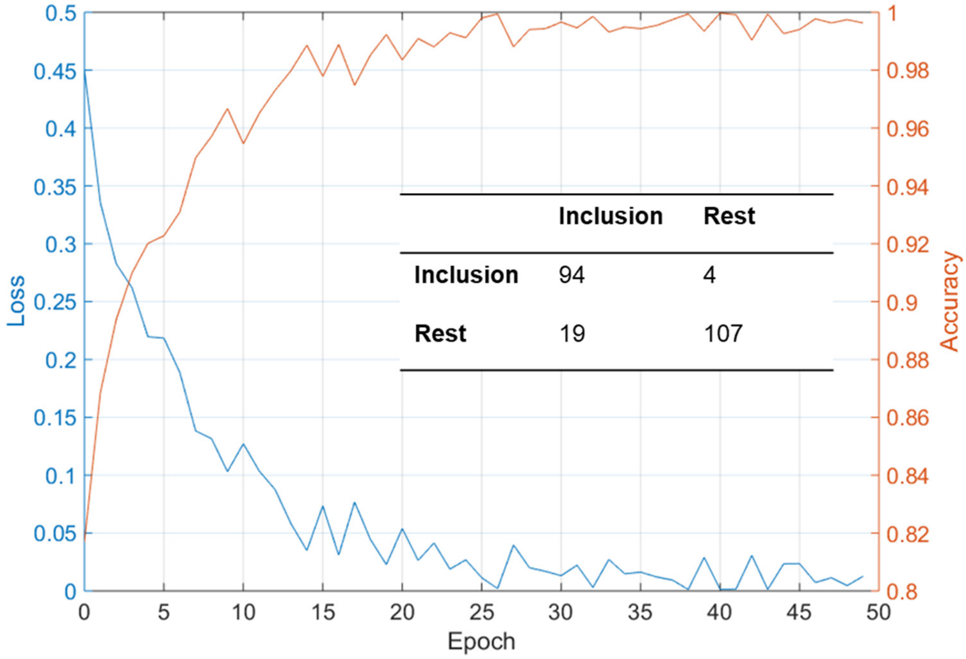

	Inclusion	Rest
**Inclusion**	94	4
**Rest**	19	107

Fig B: Accuracy and loss for the second classifier.

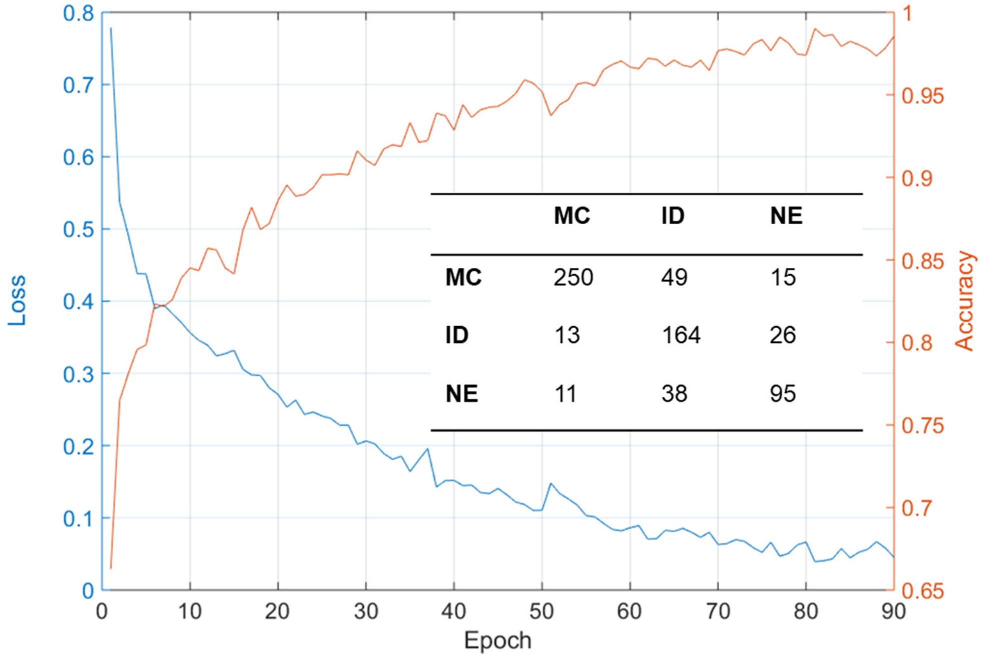

	MC	ID	NE
**MC**	250	49	15
**ID**	13	164	26
**NE**	11	38	95

Comparison of alternative networks

Table A. Accuracies of various tested network architectures for the first network.

Network	Accuracy		
	Ex-situ dataset	In-situ dataset	Total dataset
Xception	0.868	0.878	0.866
InceptionResNetV2	0.854	0.849	0.888
InceptionV3	0.901	0.863	0.915

Validation and hyperparameters

A validation set is typically used while tuning the hyperparameters of the model such as the number of hidden nodes in a network or other parameters e.g. in the pre-processing step. For this study the following parameters could be regarded as hyperparameters: The parameters of the DBScan to pre-process the images and the network architecture including the number of network nodes and layers.

The available amount of data is already one of the limiting factors of this study. Taking e.g. another 20% of the data as validation sample would only leave 60% of the original data for training which would amplify statistical fluctuations and artefacts. Hence, a separate validation sample was not used. Instead of developing and optimising custom network architectures, the CNNs used in this study are based on “standard” CNN architectures from the literature, which have e.g. been proven performant in external competitions such as the Imagenet Challenge. For the case of the DBScan, the parameters have been fixed once beforehand and are used throughout the analysis. These parameters can in principle be influenced by the electron microscope, its settings, the material and its preparation as well as other influencing factors. However, the exact choice of the parameters is not critical in this study since it is not required that all potential damage sites are found and each prospect is verified manually by an expert before the decision is made to add this to the data-sample used in the neural network analysis or discard it.
